# P-493. Decreasing Depressive Symptoms among Adults Living with HIV - United States Medical Monitoring Project (MMP), 2015-2022

**DOI:** 10.1093/ofid/ofae631.692

**Published:** 2025-01-29

**Authors:** Siobhan M O’Connor, John Weiser, Xin Yuan, Yishiow Kuo, Linda Beer

**Affiliations:** Centers for Disease Control and Prevention, Atlanta, GA; Centers for Disease Control and Prevention, Atlanta, GA; DLH Corporation, Atlanta, Georgia; DLH Corporation, Atlanta, Georgia; Centers for Disease Control and Prevention, Atlanta, GA

## Abstract

**Background:**

People with HIV (PWH) have higher rates of depression than people without HIV. We assessed trends in prevalence of depression among PWH in the United States (U.S.).

**Figure:**
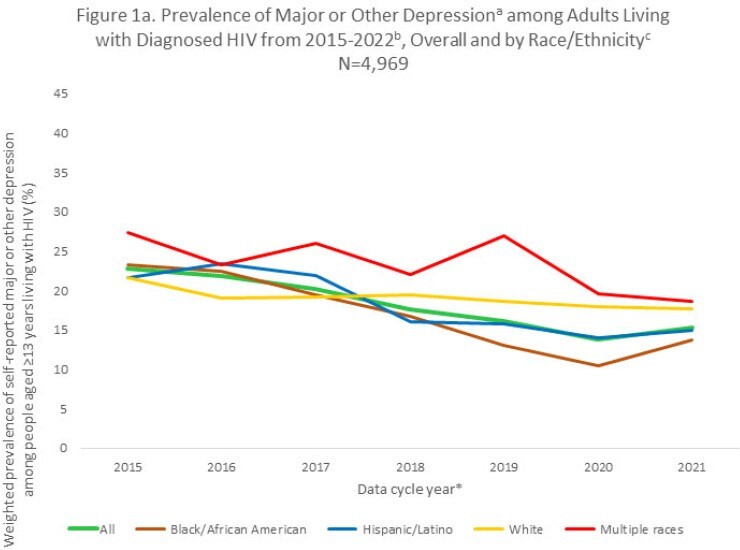

**Methods:**

We analyzed the weighted prevalence and estimated annual percentage change (EAPC), with associated 95% confidence intervals (CI), of DSM-IV criteria-defined major or other depression (depression) based on self-reported answers to Patient Health Questionnaire-8 questions from a nationally representative sample of U.S. adults with diagnosed HIV. Data were collected in 7 annual cycles (2015-2021) from June 01 of the indicated year to May 31 of the following year.

**Figure:**
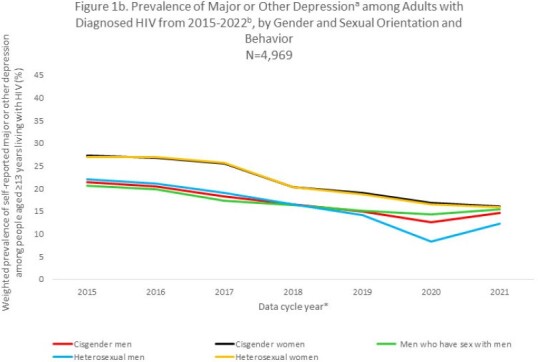

**Results:**

From the 2015 through 2021 data collection cycles, prevalence of depression symptoms decreased an average of 8% annually (EAPC = -8.0%, CI -8.1%, -7.9%). Prevalence declined across most sociodemographic groups examined. Particularly large declines were seen among PWH self-identifying as heterosexual men (EAPC = -12.4%, CI -12.6%, -12.2%) or Black/African American persons (EAPC = -11.7%, CI -11.9%, -11.6%), particularly Black/African American cisgender women (EAPC = -13.8%, CI -14.0%, -13.6%; Fig 1a-1b). In contrast, prevalence of depression increased an average of 3% (EAPC = 3.0%, CI 2.2%, 3.8%) among transgender women (measured 2016-2021 due to small 2015 sample size) and changed minimally among people who injected drugs (PWID; EAPC = 0.4, 95% CI 0.1%, 0.8%; Fig 1a-1c). Further, in 2021 high prevalence of depression persisted among adult PWH who self-identified as transgender women (27.9%), PWID (39.5%) or aged 18-24 years (24.4%); experienced food insecurity (33.1%), homelessness (29.7%) or unemployment (22.8%); or self-reported disability (27.9%).

**Figure:**
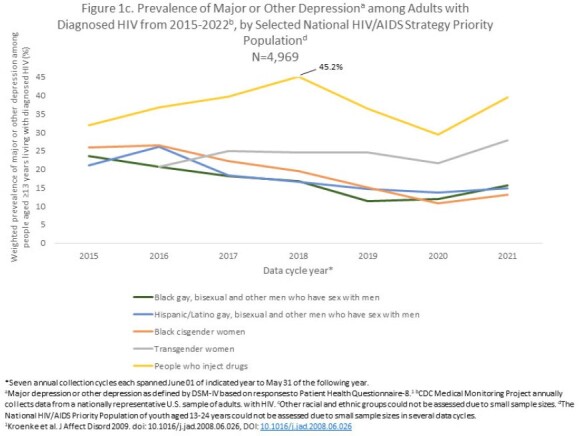

**Conclusion:**

Overall, prevalence of depression among PWH declined an average of 8% per year during 2015-2022. However, this positive outcome was not observed among certain National HIV/AIDS Strategy priority groups; depression increased in transgender women and did not change among PWID. Understanding reasons for the decline could inform interventions to further reduce depression among PWH and address unique needs of transgender women and PWID who are most affected by persisting high levels of depression.

**Disclosures:**

**All Authors**: No reported disclosures

